# The relationship between proxy agency and the medical decisions concerning pediatric patients in palliative care: a qualitative study

**DOI:** 10.1186/s12904-021-00723-4

**Published:** 2021-02-04

**Authors:** Martina Fay, Jessica Guadarrama, Tirsa Colmenares-Roa, Iraís Moreno-Licona, Ana Gabriela Cruz-Martin, Ingris Peláez-Ballestas

**Affiliations:** 1 Universidad Nacional de Rosario, Rosario City, Argentina; 2 Palliative Care Unit, Hospital Infantil de Mexico “Federico Gómez”, Mexico City, Mexico; 3 Research Department, Hospital General de Mexico “Dr. Eduardo Liceaga”, Mexico City, Mexico; 4 Graduate School in Anthropology, Universidad Nacional Autonoma de Mexico, Mexico City, Mexico; 5 Rheumatology Department, Hospital General de Mexico “Dr. Eduardo Liceaga”, Mexico City, Mexico

**Keywords:** Agency, Palliative care, Pediatric patients, Decision-making

## Abstract

**Background:**

The children’s agency and that exercised by parents and health professionals in palliative care, along with structural limitations imposed by the conditions of inequality, will provide a new perspective from medical anthropology and biomedicine to improve pediatric palliative care in complex therapeutic scenarios.

The main purpose of the study was to analyze the ways in which pediatric patients have agency in relation to their parents and palliative care (PC) professionals within the hospital setting, as well as the structural circumstances that constrain said agency.

**Method:**

A hospital ethnography (by means of non-participant observation and interviews) of the palliative care (PC) unit in a children’s hospital was conducted over the course of six months. A thematic analysis was performed using the ATLAS.ti software .

**Results:**

Thirteen cases were reconstructed of underage patients of both sexes patients together with their families; five health professionals were interviewed. The analysis identified the following 6 thematic axes, around which this article is organized: 1. The relationship between the exercise of proxy agency and the medical decisions concerning underage patients. 2. Negotiating agency and support in decision-making. 3. Child autonomy. 4. The experiences of health professionals. 5. Limitations of palliative care. 6. Bureaucratization of palliative care.

**Conclusions:**

In pediatric palliative care, agency is a process whereby different agencies intertwine: lack of pediatric patients ‘agency, the parents’ agency, the parents’ agency as representatives of their children (proxy agency), and the agency of health professionals. The concept of relational agency is proposed, defined as a set of group actions and decision-making centered around the pediatric patients’s agency and the proxy agency.

**Supplementary Information:**

The online version contains supplementary material available at 10.1186/s12904-021-00723-4.

## Background

The World Health Organization (WHO) defines palliative care (PC) for children as the “active total care of the child’s body, mind and spirit,” that also involves “giving support to the family.” Palliative care “begins when illness is diagnosed, and continues regardless of whether or not a child receives treatment directed at the disease”; it is guided by “a broad multidisciplinary approach that includes the family and makes use of available community resources” [1].

In Mexico, palliative care emerged nationally in 2013 at the behest of NGOs and academic institutions. The proposed 2014 health reform included palliative care [2] and culminated in the General Health Act of December 14, 2016, whereby it was declared mandatory [3]. Since then, major strides have been made to expand and improve palliative care. While adult palliative care has improved drastically, pediatric PC remains a challenge [2]. The pediatric palliative care is still developing and maturing in Mexico, although the recent progress has been substantial. Over the years, the main focus has been put on the hospital management of terminal and dying patients, mainly oncological, thus constantly suggesting the images of death to relatives and doctors. The situation began to slowly change, spurred by the Mexican government’s publishing of a resolution in 2014 declaring “the compulsory nature of a comprehensive palliative care management services, as well as the processes set forth in the Guide for the Comprehensive Management of Palliative Care in Pediatric Patients” [3]. This resolution marked the moment when the children suffering from incurable, progressive, and multifactorial diseases-not always oncological-were finally able to receive some overdue attention. It also helped the pediatric medical community in Mexico to adjust their approach to include patient follow-up during the course of the disease, without the limitations of the disease duration or stage.

These social, cultural, and legal changes have broadened the care framework and work panorama for pediatric palliative care as they allow interventions to improve patients’ quality of life throughout the course of the disease. In addition, the society at large is leaving behind the ingrained and erroneous belief that palliative care is only meant for dying and terminal patients, embracing the concept of care aimed at improving patients’ and relatives’ quality of life. The incumbent government’s National Development Plan 2019–2024 prioritized palliative care by taking such steps as ensuring the continuity of the process of implementing palliative care guidelines nation-wide, training health professionals especially at the primary care level, and including palliative care in undergraduate medical schools curricula [4].

Age is another relevant aspect for pediatric palliative care in Mexico, which sets the age of majority at 18 years. However, PC health professionals and particularly physicians and parents tend to consider the opinion of minors aged 8–18 years. For children under 8 years, the wishes and preferences of the parents or guardians are automatically considered above those of their children [5].

A non-systematic review of the biomedical and social literature revealed a scarcity of publications with an anthropological approach to palliative care in children; rather, most publications are focused on bioethical aspects and decision-making [6].

A prior qualitative study of pediatric PC departments in Mexico working with adolescents with cancer identified certain barriers to decision-making, such as communication, paternalistic attitudes exhibited by the oncologists, therapeutic futility, and limitations for adequate care. However, the study focuses on PC in pediatric oncology and is limited to the end of life stage [7].

Our theoretical reflection draws on the social anthropology of childhood, which regards children as social actors and main protagonists at the center of interactions and relationships, which brings about their agency [8, 9].

The concept of agency, as put forth by Gramsci, refers to the ability to act both individually and collectively in the societal transformation brought about by the individual, and to how the individual is transformed by social and political structures [10]. According to Giddens, agency is exercised by an individual as an author who can act in a variety of ways [11]. Both authors focus on action as transformation, on decision-making, which can vary depending on the events and contexts in which the individual needs to make decisions that transform his or her actions as individuals and part of a collective, and, in turn, as the collectivity transforms them as individuals. In this study, therefore, the pediatric patients exercise their agency in relation to their family and to the health professionals in a social context. This particular social context sees some structural conditions come into play that modify this collective agency, such as the health care system and the living conditions of the pediatric patients and their family. Understanding agency of the individual patients (children’s agency) and that exercised by parents and health professionals in complex therapeutic settings, along with structural limitations imposed by the conditions of health inequality, will provide a new perspective from medical anthropology and biomedicine to improve pediatric palliative care in complex therapeutic scenarios.

## Objectives

1. Describe and analyze the ways in which pediatric patients exercise agency in relation to their parents and palliative care professionals within the hospital setting.
2. Contextualize and analyze the impact of the agency exercised by the triad comprising a pediatric patient and parents and caregivers on the one hand, and PC professional on the other, considering both the barriers and enablers to public palliative care.

## Methods

This study has a qualitative design. For this study, hospital ethnography was used to collect the data [12, 13], which included non-participant observations and semi-structured interviews with all health professionals in the Palliative Care Unit of the Pediatric Hospital. Given the delicate and sensitive situation they were going through, patients and relatives were not interviewed. However, they allowed an anthropologist (MF) to be present during consultations. Each patient’s clinical health record and psycho-social background were also analyzed, as well as those of their families.

This ethnography was performed (MF) over the course of six months in 2019. Encounters between patients, family members and health professionals were observed under a variety of circumstances, such as individual consultations in the PC unit office, group grief workshops for parents, and in-hospital doctor’s visits. Convenience sampling of the observations was used, i.e. all cases that arose Monday through Friday in which the anthropologist was present during visits and consultations conducted by the PC team. Health professionals from the PC service were present during the interviews. Field notes were made during the visits, consultation and interview. Minors under 16 years were not interviewed.

Each PC health professional was interviewed (MF) face-to-face privately with a guide prepared by an interdisciplinary group of physicians, pediatricians, ethicists, and medical anthropologists, to explore aspects related to the objectives of the study (Additional file 1. Interview guide). The interviews lasted a median of one hour and were audio-recorded for subsequent transcription and analysis.

The cases presented in this article build on information from observations (Additional file 2. Observation guide), interviews, as well as patients’ medical, psychological, and social work records, providing context and deepening our understanding of each case, which in turn helped to shape the ethnography (including field notes). After structuring the cases picked for the analysis, we proceeded to conduct a thematic analysis of the collected data. Version 7 of the ATLAS.ti software suite was used for the thematic analysis [14]. The analyses were performed independently by three medical anthropologists (IPB, TCR and ACM) and were reviewed for convergence (of the identified themes; the thematic analysis was subsequently reviewed jointly with two PC specialists to reach an agreement regarding the interpretation needed for data triangulation. All researchers were female.

## Results

Thirteen cases of patients and their relatives were reconstructed by non-participant observation and record examination (see Table 1). Five narratives of PC unit professionals were reconstructed from interviews and in-hospital observation, of these all were female, age group was 25 to 40 year; two psychologist (Ps), one pediatrician (P), one general physician (GP) and one social worker (SW). For work experience in PC, one (GP) with 10 months, three (Ps and SW) with 2 year and one (P) had up to five years. No body refused to participate or dropped out.

**Table 1:** Patient cases

Case	Setting	Family	Social Security (SS) & support services	Observations	Medical Diagnosis
B	Urban	Extense family (6 members)	With SS & Foundation support services	The mother and the grandmother in psychology and social work meetings	Meningoencephalitis viral etiology (chicken pox) Severe consequences & focal epilepsy by management.
C^a^	Urban	Nuclear family (four members) Mother: head of the family	Without SS nor support services	The mother in social work meetings.	Malignant brain tumor. Resection and brain swelling.
A	Urban	Extense family (10 members)	With SS & Foundation support services	The mother and the patient in psychology meetings.	Primary Embryonal Synovial Sarcoma in pelvis, with metastasis in abdominal peritoneum & lungs.
J^a^	Urban	Extense family (8 members)	Without SS nor support services	The mother and the father in psychology meetings.	Gastroschisis with first intention closure
G	Rural	Nuclear family (5 members)	With SS	The mother in pediatric and social work meetings.	Tricuspid atresia
MB	Urban	Nuclear family (4 members)	Without SS, Toddler’s milk support.	Parents workshop with the psychologist	Pierre Lobin syndrome. Cornelia de Lange syndrome. Phocomelia. Pulmonary hypertension. Poor swallowing mechanics. Central apnea.
R	Urban	Nuclear family (5 members)	With SS & social support service	The mother and the patient in psychology and social work meetings.	Brain tumor
Y	Urban	Nuclear family (5 members).	With SS		Regressive syndrome. Bilateral hypoacousis.
J	Urban	Extense family (11 members)	With SS	The father in psychology meeting	Non- convulsive status epilepticus. Structural epilepsy & corpus callosum hypoplasia. Congenital laryngomalacia.
CN^a^	Urban	Nuclear family (4 members). (The family is identified as indigenous at least in part)	With SS	The mother and the patient in psychology and social work meetings.	Congenital Glioblastoma multiforme .
S	Urban	Nuclear family (6 members).	With SS	The mother in psychology meetings and social work meetings.	Congenital neutropenia
M	Urban	Nuclear family (5 members).	With SS	The mother and the father in psychology meetings.	Lung deficiency & heart disease.
JP	Urban	Nuclear family (3 members).		The mother and the patient in psychology meetings.	Hipotonic syndrome, breathing problems

^a^ Children who passed away before the article publication

The hospital setting where the research took place is described, together with the history of the Palliative Care Unit. The common themes that have been identified are represented by selected excerpts picked from the field diary and the interviews.

This research was carried out in a highly specialized pediatric hospital that especially caters to socially and economically disadvantaged pediatric patients from central Mexico.

### Palliative care in an everyday context

The Palliative Care Unit (PCU) was first conformed by a group of pediatricians interested in palliative care, five years before the unit officially appeared on the hospital’s organization chart. Out of their concern for prolonged hospital stays of cancer patients, a project arose which sought to reduce the length of stay, strengthen community health care networks, and thus develop child/family care circuits connecting hospitals and households. This change proved to be cost-effective, so the hospital began the process of setting up the Palliative Care Unit, which officially opened a year later, in October 2018. In the first year (2018–2019) the unit provided care to 250 patients and their families, with a 50% rate of home care services [15].

The PCU team comprises a pediatrician (P) with a master’s degree in palliative care and bioethics, and four non-permanent health professionals: a medical intern (MI), two psychologists (Ps) specializing in family therapy, and a social worker (SW). To quote the head of unit, “this configuration of the team is ideal for a highly specialized group,” as the team is focused on providing care as well as doing research into the social and economic aspects of palliative care. The Palliative Care Unit is located in a single small office shared by all team members; home care is a priority, so visits and telephone follow-ups are a key part of the team’s activities.

Daily life in the PCU can be overwhelming. While all team members interviewed had a hard time describing a typical day, they all agreed that it varied a great deal, with multiple duties ranging from recording new cases, to visiting the hospital admissions unit, administering interviews with parents or caregivers, providing help to navigate post-death formalities, and offering grief workshops for parents and relatives. As described by three of the PCU team members, their department of only five health professionals deals with a heavy workload in a large hospital specializing in providing complex medical care. Their work helps build bridges between pediatric patients, attending physicians, and children’s families, in addition to creating support networks for the problems that families face in the home and hospital setting.

We identified the following central themes from the analysis of the cases of the patients/families, and the health professional interviews: 1. The relationship between the exercise of proxy agency and the medical decisions concerning underage patients. 2. Negotiating agency and supporting decision-making. 3. Child autonomy. 4. Experiences of health professionals. 5. Limitations of palliative care. 6. Bureaucratization of palliative care.

#### The relationship between proxy agency and the medical decisions concerning underage patients

In a hospital setting, the voice of the parents replaces that of their children. In the observed cases, when the child is very young or when her abilities to communicate have been compromised, parents relay what their child is going through or feeling, letting the physician know whether she is in pain or tired, whether she eats, and so on. They claim that they understand what is happening with their children to pass this information on to the health professionals. The minors’ ability to act in the world is limited by their age and life-threatening illness, which is why that agency is transferred to, or assumed by, parents, in what we call proxy agency:

“He (J) doesn’t say anything, he doesn’t say that he’s in pain. We know him and we know what he does and doesn’t like. That’s why when they do something to him that I know he doesn’t like I have to tell them that” (case of J).

When the child does not have the ability to communicate, palliative care professionals ask parents directly instead of asking the child. Health professionals bear in mind the child’s limited ability to communicate due to their health condition by asking easy questions about daily life or other matters related to their health:

G asks A what he wants to do. A’s mother says he wants to keep going to school and going out. G tells A and his mother that it is very important that they reach an agreement that both find acceptable. At this point, A’s mother repeats that she does not want him to go out alone by himself. Finally, they agree that A can continue going to school in the morning as long as he gets his friends to walk him back from school (case of A).

As far as the decisions about treatment or those with far-reaching implications, it is parents who communicate their wishes related to the future of their children.

Both [parents] say that they want their child to continue having all relevant and available medical procedures (case of O).

“Many parents will tell you, ‘my girl doesn’t want anything’ so I respect that. There are also many parents who keep saying, ‘no, I’m not giving up, I see that my boy carries on fighting so I am not going to give up,’ or ‘if she’s not surrendering, neither will I and I will keep at it.’ So obviously the child has no way of expressing her autonomy... [In other cases] it can be seen more in older children that when they say ‘I give up, I don’t want this anymore,’ the parents follow suit by saying ‘okay, not anymore’” (GP).

The children’s ability to act, that is, execute agency and express it through decision-making, is assumed by their parents, who express what they want for their child rather than what the actual child wants. The professionals we interviewed report that the child’s opinion is considered, although it is the parental opinion that will matter when making important decisions. It is a kind of proxy agency.

“When the worst is about to happen, a seven or eight-year-old kid will tell you what she wants, and more so if they are teenagers. It becomes very clear that you have to listen not only to the voice of the parents, but also that of the children. Everything considered, it is the parents who make the final decision, but they can’t force their child, the child’s opinion has a certain weight to it already, and it’s our responsibility to give them options for a good quality of life which don’t conflict with the decisions they make” (P).

#### Negotiating agency and supporting decision-making

Decisions about treatment and its consequences are arrived at by the attending physicians and relayed to the parents, who are not always ready to make their decision immediately. In these circumstances, the doctors engage the support of the PC team members as mediators to exercise the agency (proxy and parental) in decision-making from the perspective of the child’s quality of life.

With doctors, it [the communication] has to be very close, because as palliative care workers we support the physicians [from other departments of the hospital] in managing their patients … As far as the parents go, you need to help them make treatment decisions that their doctor proposes. The parents will often be the first to say, ‘I don’t want [the child] to be given anything if you can’t promise he will be cured.’ And the doctors, especially oncologists, would often say, ‘there is a 1% chance that he will carry on, that he will live for three more years.’ Yes, but being a part of that 1% entails a lot of suffering and it’s not a given that the child will fall into that 1%... but [we the PC team ask], what do you and your child want? That is our work as a link between parents and doctors (GP).

#### Child autonomy

For health professionals, reaching autonomy is a process connected to personal development:

“… Whether they will grow up to be autonomous, to have autonomy, depends on how they’re brought up, right? [In] the development process, sick children often have some pros and some cons, perhaps parents can be too caring, and their ability to accept or reject these decisions, as with adolescents” (Ps).

The agency of children and the family and, therefore, the decision-making in the palliative care setting are guided by the makeup of the PC department, the experience of the PC group, and the structural aspects of care, especially such elements as lack of staffing and shared space for providing private care to parents and children, as this affects the main objective of palliative care—providing a humanistic care for children and their families to improve their quality of life.

#### Experiences of the health professionals

The experience of working in the PCU was mixed among the team. One constant that was true for all team members was their desire to help patients and their families in distressing situations, as well as to advance the professional’s own learning.

“[...] Because here I learned what it means to see the benefits, to understand what is better for the patient … That is something I didn’t acknowledge before, but now I say yes, I can see it” (Ps).

“The impact that having worked here in palliative care had on me, on the team, the group, was that it has made me more humane, more empathetic … I have seen death, poverty, families, what families are really like …” (Ps).

#### Barriers and enables to palliative care

**Fig. 1:**
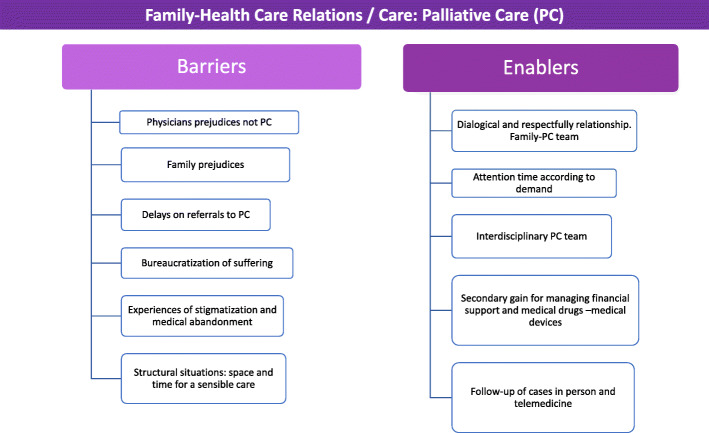
Barriers and facilitators that affect the relationship between family members and health professionals in the palliative care service of a children’s hospital in Mexico City, which were identified through interviews and non-participant observations

The aspects limiting the provision of care by health professionals are: communication with the parents; the relationship with the attending physicians, especially oncologists; understaffing in the PCU; addressing families’ living conditions such as the lack of financial resources; domestic violence; or the need to migrate to the city in search of quality palliative care (Fig. 1).

##### Communicating with parents: “Whatever you do, don’t lie”

“[Communication is hard] because we’re not supposed to tell the kids their diagnosis. Yes, there’s a barrier between parents and children because the parents don’t want to tell them neither the prognosis nor the type of the disease the kids have, not even that they might die, to explain that this is a possibility. The kids of course realize that and know everything perfectly, but they’re also afraid to ask because their parents aren’t telling them anything. To me lying to children is worse, to tell them ‘you’re going to be fine, you’re going to get well and leave [the hospital]’ when you actually know the kid might be able to leave not because she’s in any pain, but because there’s no cure … Parents must be spoken to; painful as it might be, you need to tell them the truth because it’s the only way for them to face the problem. If they don’t know what they’re dealing with, they can’t face it … you make them do a reality check” (P).

##### Room

The consultations in the PC unit offer little privacy to the patients: the place is full of health professionals and the interruptions are frequent, from phone calls, extraneous noises, other professionals and patients coming and going, or maintenance activities. The patients do not seem to find so much interference disruptive; however, the team members and the anthropologist still believe that the lack of room is an issue, as this kind of work requires a more private setting.

##### Working together with other hospital health professionals

Working relationship with other health professionals is ambivalent. On the one hand, many physicians consider their unfamiliarity with the role of palliative care as an important omission, along with the associated taboos, or the perception that palliative care is something ‘new.’

“...we still need... people still need to link palliative care with quality of life because always, that is, not even just patients, many doctors, many psychologists say ‘palliative care means [they’re] agonizing, that there’s no cure’” (Ps).

“...because I feel that much of the medical taboos around palliative care comes from that... from that feeling that you can’t help any more and you feel powerless and frustrated...” (GP).

Conversely, doctors also mention the need for the palliative care workers to support or accompany hospital health professionals in navigating through the difficult stages of care for children and their parents.

“And with the professionals, with those who are around them, I believe that we [PC team] should also help them, because it’s a difficult process for them too … it’s a grieving process for them … we should care for them too, but we don’t...” (Ps).

##### Delay in referring to PC

Late referral to the PCU has many causes including misconceptions, poor knowledge and existing taboos towards PC.

“Educating hospital staff has been a tall order, basically because many doctors believe that palliative care begins when the child is dying, and they continue to refer patients to me who die within hours. This is wrong, this is not right... We’ve been having a hard time establishing contact [with the attending physicians]... to make them see the importance of early referral” (P).

##### Structural limits to palliative care

According to team members, the inadequate provision of palliative care is caused by a combination of various factors: limited funding in public hospitals; poor sustainability performance of the non-institutional support networks (specifically, NGOs that depend on the flow of donations, which is not constant due to an underdeveloped donation culture in Mexico); and the fact that the unit’s creation is recent.

“Because our patients are very poor, exceedingly poor, we always have to be prepared to help and we have to work with charities, and also look for... I think if we had a shelter, a.... No, not a shelter, if we had a constant flow of funding, we could have some improvements...” (Ps).

Reaching out to the primary health care providers and NGOs that can support patients in conjunction with the PCU is the greatest challenge for the PC team.

“...discharging [the children], which is our main objective, so they can get home. But sometimes the services... the supplies, like a fan that is super expensive or insulin pumps, are what stop us from achieving a certain objective, that is to try to give the best quality of life possible within the hospital” (SW).

“There are no home visits, so the patient has to come to the hospital for care. How am I doing it right now? By phone. But speaking over the phone is not the same as when a doctor is going to see you [in person], to tell you that you are doing well... the challenge is where even that [phone service] is not available [in other regions of the country] in the poorest areas” (P).

Other structural family-related aspects are lack of financial resources for health care, unhealthy family dynamics (such as domestic violence and alcoholism), and the distance to the palliative care centers.

These limitations notwithstanding, we also identified certain facilitating factors to palliative care: a relationship based on respect and dialogue; an empathetic care that can meet the needs of the pediatric patients and their families; a committed interdisciplinary group (as could be seen from the experiences shared by the members of the PC team); out-of-hospital networks aimed at solving certain structural problems of the families and the health care system; and ongoing support using telemedicine.

### Bureaucratization of palliative care

As part of the hospital procedures in place for patients with complex, life-threatening diseases, parents must sign a do-not-attempt resuscitation order for extreme cases, known as Cardiopulmonary Resuscitation 3 (CPR3). When children are admitted to the hospital, the doctors in different hospital areas push the parents to sign the order as quickly as possible, which is perceived as a lack of empathy by the relatives in the face of pain and suffering of their children; therefore, relatives are reluctant to sign. The CPR3 order is a point of contention between the palliative care unit and other medical areas. The latter oversee drafting the order and storing it in the patient’s clinical record, while the PCU maintains a document called ‘consent under information’ (CBI for its Spanish acronym), which includes the following records: diagnosis, prognosis, disease characteristics, and measures to provide care and avoid invasive therapeutic procedures. The CBI includes CPR3 and the possibility for the parents to revoke the do-not-resuscitate order at any time; the two latter aspects are emphasized to the relatives. The way that explaining and signing works in the PCU is also different. While in the hospital admissions office, parents are urged to sign CPR3 as soon as possible out of the regulatory requirements. In contrast, at the PCU, where signing the order is equally important, it is considered crucial to prepare the parents psychologically, walk them through the document, and give them time to consider it before signing. As it can be seen, these are two different approaches to the same sensitive aspect of care. Palliative care professionals find it ideal that they get involved in the process as early possible.

“When the boy entered a critical stage, the doctor [from the oncology department] insisted that he [the father] sign the form; he and his wife were crying, both were in a really bad way and the doctor just kept going about the [CPR3] form. He [the father] later said had a fight with his wife... He said he made her feel guilty for signing that paper. He said that he apologized to her after” (Case of J).

“The father says that the doctor made him sign the RCP3 form, that it hurt, but that she was in a lot of suffering” (Case of M).

Physicians who are in charge of signing the order consider the process differently depending on whether they are from the PCU or some other department:

“But the relationship between the ‘palliatives’ and other doctors is that [the latter] see it as a legal relationship, as this legal thing... it’s always hard because it’s a very legal idea to them, as something that ‘if I don’t do all of it [the procedure], there will be trouble’ …” (GP).

The CBI document, signed by doctors and parents, becomes a manifestation of the parents exercising the proxy agency, since the children cannot make decisions concerning their bodies and wellbeing; however, the parents themselves exercise it under the influence of the medical structure:

“It [the CBI document] is signed by the attending physician, the PCU doctor and by mom and dad accepting those conditions. At any rate, that document is revocable... The children as they are, precisely because they are minors, can’t be signing something, right? That is, but yes, it is about working with the parents so that the parents update that paper, if necessary” (SW).

## Discussion

The relational agency observed in this study amalgamates the agencies of children, of the parents as caregivers (proxy agency), and of the palliative care psychologists as participants of a decision-making process that aims to enhance the quality of life and dignity of the patients. The proxy agency is agency exercised by the parents aided by the PC professionals to support the decisions made by the child—if the child is physically capable of expressing agency—or by the parents themselves, who perceive the needs of their children as related to the experience and technical expertise of the PC team members. This relational agency cannot be understood or explained without considering the conditions under which it is exercised, i.e. a palliative care setting in a hospital, with limited family resources, in a cultural context admitting a variety of interpretations as to how to deal with a sick child with the possibility of near death, all taking place within palliative care program that is just getting off the ground nationally.

The health agency refers to a pediatric patients ‘s ability to recognize life choices, conditioned by his or her age, gender, place in the family, peers, and the emotional and socioeconomic status of his/her parents/caregivers to express and execute power in making decisions related to their well-being.

Magistris differentiates between the agency of children and that of adults, stating that the former “(...) occurs in a particular structural context, which is a subordinate minority action framework” (9), while the latter enjoys greater recognition and legitimacy, thus reducing or nullifying the development of the children’s agency; the author refers to it as ‘adultcentrism’ [9]. The perspective proposed by Magistris has little application in this study. This limitation would seem to correspond to participation through silence, which can be interpreted as part of the age, severity of their health condition, child’s behavior in the face of pain, and the imminence of death; rather than denying him the right to name and share his experience and therefore participate in the decision-making, this is an expression of resistance, of an impossibility to share [16].

The relationship between the tryad parents-children/palliative care professionals is connected to truthfully informing the pediatric patient, as well as to the patient’s and the patient’s family’s right to know the truth. Yet, as evidenced in this study, giving information to the child meets more resistance from parents than from palliative care professionals. This aspect is one of the most demanding parts of the process; it concludes when the appropriate wording is found. This is in agreement with the findings reported by several other authors who describe that the relationship of medical workers and parents with the child correlates with the development of the child’s autonomy [17–20]. Here we would like to place a special emphasis on agency and the participatory decision-making within the triad pediatric patient/family/health professionals.

Cicero-Oneto et al. [7] describe the predominance of a paternalistic approach within the relationship between the oncologists, the parents and the child. Moreover, oncologists perceive that parents have trouble understanding information and especially making decisions (this situation is similar to the one described by the participants of our study). Another point related to the barriers to care observed and described by the psychologists is the attitude exhibited by the attending physicians towards the palliative care unit: while the unit is regarded as a place to which the family and the child are referred when there are no more therapeutic options left, the physicians believe that hospitals should have a specific place with adequate conditions to deal with the issues at the end of life. This situation is described by the authors [7].

The experience of health professionals is described in an environment of multifaceted emotional tension, of concern for the well-being of the child and their family in their socio-economic context [21]., in a study carried out in Japan, describe the dilemmas faced by pediatricians in children’s palliative care. They are focused on decision-making informed more by internal than external values, and aim to act in the child’s best interest to find the best therapeutic options and help the family to provide adequate care at home. These dilemmas were not observed in this study; rather, the difficulties faced by the members of the PC team revolve around communication barriers between them and the attending physicians, lack of space, and financial hardship of the families they serve, making clear the socio-cultural contrast between countries like Japan and Mexico with different health care systems [21].

The parents’ experience with the bureaucratic routines such as the signing of the do-not-resuscitate order has been subject of controversy among psychologists worldwide; for Clark and Duzinski [22], such an order should be an informed consent based on a process whereby the physician informs the parents about risks and benefits of treatment so they can make a decision. The PC psychologists who participated in this study agree with this proposal regarding the CPR3 [22–25].

### Limitations

We identified important family themes, such as domestic violence, parental risk behavior, and the conceptualization of death. Although they were not explored in depth, these are important themes for future studies.

On a more theoretical level, this study invites to consider two very close concepts: autonomy and agency. Both concepts pose enormous challenges when applied to underage individuals (children and adolescents). However, while agency comes about and is exercised in a social context, autonomy occurs in the ethical and legal context. An in-depth consideration of these concepts as applied to the area of palliative care in children would go a long way, but this falls beyond the scope of this article.

One more limitation of our study is the non-participation of children in the interviews, as this was deemed inconsiderate; however, their opinions in non-participant observations of consultations and visits to hospital wards were observed and captured.

## Conclusions

Agency of the actors within the pediatric palliative care setting is a process whereby different agencies come into play. That is, the parents’ own agency, their agency as representatives of their children (proxy agency), and the agency of health professionals. This plexus of agencies and decision-making appears in the hospital context and is configured through family relationships (with their difficulties, such as emotional and economic problems), the experiences of health professionals, and the structural problems of a palliative care department, envisioned as a humanistic proposal to care for the pediatric population in Mexico. The notion of relational agency is proposed as a set of actions and group decision-making centered around the children’s agency and proxy agency as the main concept of the palliative care in pediatrics.

## Supplementary Information

**Additional file 1.**

**Additional file 2.**

## Data Availability

All data generated or analyzed during this study are included in this published article.

## Abbreviations

PC: Palliative care
CPR3: Cardiopulmonary Resuscitation 3
WHO: World Health Organization
GP: General physician
SW: Social Worker
Ps: Psychologist
PCU: Palliative Care Unit

## References

[1] World Health Organization. Palliative Care. 2020. https://www.who.int/news-room/fact-sheets/detail/palliative-care. Accessed: 20 Feb 2020.

[2] Mexico Human Rights Watch (2014). Cuidar cuando no es posible curar.

[3] Diario Oficial de la Federación. ACUERDO por el que se declara la obligatoriedad de los esquemas de manejo integral de cuidados paliativos, así como los procesos señalados en la Guía del Manejo Integral de Cuidados Paliativos en el Paciente Pediátrico. 2016. http://www.dof.gob.mx/nota_detalle.php?codigo=5465444&fecha=14/12/2016. Accessed 22 Apr 2020.

[4] Santos-Preciado JI, Anaya-Nuñez R, García-Moreno J. El Consejo de Salubridad General (CSG) y los Cuidados Paliativos. Consejo de Salubridad General. www.csg.gob.mx. Accessed 11 Jan 2021.

[5] Guadarrama-Orozco J, Garduño-Espinosa J, Vargas-Lopez G, Viesca-Treviño C (2015). Informed consent and parental refusal to medical treatment in childhood. The threshold of medical and social tolerance. Part I. Bol Med Infant Mex.

[6] Ghirotto L, Busani E, Salvati M, Di Marco V, Caldarelli V, Artioli G (2019). Researching children’s perspectives in pediatric palliative care: a systematic review and meta10 summary of qualitative research. Palliative Supportive Care.

[7] Cicero-Oneto CE, Valdez-Martinez E, Bedolla M (2017). Decision-making on therapeutic futility in Mexican adolescents with cancer: a qualitative study. BMC Med Ethics.

[8] Wainer R (2008). Hacia una Antropología del morir-entre-nosotros o cómo entender el afecto en el final de la vida en niñ@s. IX Congreso Argentino de Antropología Social.

[9] Magistris G. La construcción del 'niño como sujeto de derechos' y la agencia infantil en cuestión. Journal de Ciencias Sociales. Revista Académica de la Facultad de Ciencias Sociales de la Universidad de Palermo 2018; 6(11):6–28.

[10] Pizza G (2005). Antonio Gramsci y la antropología médica contemporánea. Hegemonía, “capacidad de actuar” (agency) y transformaciones de la persona. Revista de Antropología Social.

[11] Giddens A (1984). The constitution of society: outline of the theory of structuration.

[12] van der Geest S, Finkler K (2004). Hospital ethnography: introduction. Soc Sci Med.

[13] Finkler K (2004). Biomedicine globalized and localized: western medical practices in an outpatient clinic of a Mexican hospital. Soc Sci Med.

[14] Nowell L, Norris J, White D, Moules N (2017). Thematic analysis: striving to meet the trustworthiness criteria. Int J Qual Methods.

[15] Martínez A (2019). Departamento de Cuidados Paliativos, una nueva arista en el HIMFG. Ixtlilton La revista del HIMFG.

[16] Heredia CR (2019). ¿Cómo es el dolor? Indagaciones médicas, registros y etiologías del dolor en cuidados paliativos pediátricos. Cuadernos de Antropología Social.

[17] Wangmo T, De Clercq E, Ruhe K, Beck-Popovic M, Rischewski J, Angst R, Ansari M, Elger B (2017). Better to know than to imagine: including children in their health care. AJOB Empirical Bioethics.

[18] Martakis K, Brand H, Schroder-Back P (2018). Developing child autonomy in pediatric healthcare: towards an ethical model. Arch Argent Pediatr.

[19] Bluebond-Langner M, Belasco JB, De Mesquita Wander M (2010). “I want to live, until I don’t want to live anymore”: involving children with life-threatening and life shortening illnesses in decision making about care and treatment. Nurs Clin N Am.

[20] Coyne I, O’Mathuna PD, Gibson F, Shields L, Sheaf G. Interventions for promoting participation in shared decision-making for children with cancer. Cochrane Database Syst Rev. 2016;11(11):CD008970. 10.1002/14651858.CD008970.pub3. 10.1002/14651858.CD008970.pub3 PMC6734120 27898175

[21] Sasazuki M, Sakai Y, Kira R, Toda N, Ichimiya Y, Akamine S, Torio M, Ishizaki Y, Sanefuji M, Narama M, Itai K, Hara T, Takada H, Kizawa Y, Ohga S (2019). Decision-making dilemmas of paediatricians: a qualitative study in Japan. BMJ Open.

[22] Clark JD, Dudzinski DM (2013). The culture of Dysthanasia: attempting CPR in terminal ill children. Pediatrics..

[23] Hsiao J, Evan E, Zelter L (2007). Parent and child perspectives on physician communication in pediatric palliative care. Palliative Supportive Care..

[24] Tates K, Meeuwesen L (2001). Doctor-parent-child communication. A (re) view of the literature. Soc Sci Med.

[25] World Health Organization (2013). Strengthening the doctor–patient relationship. A framework for action.

