# Supplementary material for: The relationship between proxy agency and the medical decisions concerning pediatric patients in palliative care: a qualitative study
Source: BMC Palliat Care. 2021 Feb 4;20:27. doi: 10.1186/s12904-021-00723-4 (PMC7863456; doi:10.1186/s12904-021-00723-4)
Supplement: Supplementary file 1 — Additional file 1. [file 12904_2021_723_MOESM1_ESM.docx]

**INTERVIEW GUIDE TO HEALTH PROFESSIONALS IN PALLIATIVE CARE**

**Thems to explore**

**1. Introduction**

1.1Personal issues: Gender, age, academic preparation, position, and rol in the hospital, , 1.2 Approved to participation

1.3 Select a pseudonym.

**2. Journey in the daylife in the Palliative Care Unit**

2.1 Describe a "normal" day in the Palliative Care unit

2.1. Describe how the Palliative Care unit Works

2.2 Describe barriers and enablers in the Palliative Care unit

3**. Describe the career path in palliative care**

3.1. Experience: Since when, because they chose the topic of palliative care in children and how they were integrated into Hospital's work team.

3.2. Impact of this type of work on the professional trajectory and personal life.

3.3 What are the strategies they use or used to deal with the impact of the work

3.4 What are the limits of palliative care in cases and when cases refer to other hospital services

**4. Describe the population to which the service or counseling**

4.1. Who should receive palliative care counseling

4.2 Describe the counseling process (from the call of the service requesting it until the end of the case)

**5. Describe your relationship with the populations you care for (patient and family) and other health professionals**

5.1. Decision-making ability for patients to make decisions

**6. History of the Palliative Care Unit and recommendations for improving the Palliative Care Unit.**
