# Supplementary material for: The relationship between proxy agency and the medical decisions concerning pediatric patients in palliative care: a qualitative study
Source: BMC Palliat Care. 2021 Feb 4;20:27. doi: 10.1186/s12904-021-00723-4 (PMC7863456; doi:10.1186/s12904-021-00723-4)
Supplement: Supplementary file 2 — Additional file 2. [file 12904_2021_723_MOESM2_ESM.docx]

**Guide to Observing at Hospital Context of Palliative Care in Children and** **Adolescents**

T**hem 1. Children's agency and decision-making**

1.1Barriers/difficulties in exercising agency and decision-making

1.2Conflict and decision-making

1.3 Relationship between parents/professionals and children and decision-making

1.4 Participation of children, family and health professionals in palliative care in decision-making

**Them 2. Suffering**

2.1 How children lead their lives with the diagnosis they have

2.3 How their parents go through their lives with their children's diagnosis

2.4 Which metaphors they use (parents, children, professionals) to talk about a disease

2.5 Is there talk of death? If so, what metaphors are used to refer to it?

2.6 How children live their suffering

**Them 3. Autonomy**

3.1 Children's autonomy and their treatment

3.2 Are gender roles related in the autonomy?

3.3.What metaphors are used to discuss a condition with children?
